# High-throughput bacterial SNP typing identifies distinct clusters of *Salmonella *Typhi causing typhoid in Nepalese children

**DOI:** 10.1186/1471-2334-10-144

**Published:** 2010-05-31

**Authors:** Kathryn E Holt, Stephen Baker, Sabina Dongol, Buddha Basnyat, Neelam Adhikari, Stephen Thorson, Anoop S Pulickal, Yajun Song, Julian Parkhill, Jeremy J Farrar, David R Murdoch, Dominic F Kelly, Andrew J Pollard, Gordon Dougan

**Affiliations:** 1 Wellcome Trust Sanger Institute, Hinxton, Cambridge, CB10 1SA, UK; 2 Department of Microbiology and Immunology, University of Melbourne, Royal Parade, Melbourne, 3010, Australia; 3 Wellcome Trust Major Overseas Programme & Oxford University Clinical Research Unit, Hospital for Tropical Diseases, Ho Chi Minh City, Viet Nam; 4 Centre for Tropical Medicine, Nuffield Department of Clinical Medicine, University of Oxford, Oxford, OX3 7LJ, UK; 5 Oxford University Clinical Research Unit - Nepal, Patan Hospital, Kathmandu, Nepal; 6 Oxford Vaccine Group, Department of Paediatrics, University of Oxford, OX3 9DU, UK; 7 Environmental Research Institute, University College Cork, Lee Road, Cork, Ireland; 8 Department of Pathology, University of Otago, Christchurch, New Zealand; 9 Canterbury Health Laboratories, Christchurch, New Zealand

## Abstract

**Background:**

*Salmonella *Typhi (*S*. Typhi) causes typhoid fever, which remains an important public health issue in many developing countries. Kathmandu, the capital of Nepal, is an area of high incidence and the pediatric population appears to be at high risk of exposure and infection.

**Methods:**

We recently defined the population structure of *S*. Typhi, using new sequencing technologies to identify nearly 2,000 single nucleotide polymorphisms (SNPs) that can be used as unequivocal phylogenetic markers. Here we have used the GoldenGate (Illumina) platform to simultaneously type 1,500 of these SNPs in 62 *S*. Typhi isolates causing severe typhoid in children admitted to Patan Hospital in Kathmandu.

**Results:**

Eight distinct *S*. Typhi haplotypes were identified during the 20-month study period, with 68% of isolates belonging to a subclone of the previously defined H58 *S*. Typhi. This subclone was closely associated with resistance to nalidixic acid, with all isolates from this group demonstrating a resistant phenotype and harbouring the same resistance-associated SNP in GyrA (Phe83). A secondary clone, comprising 19% of isolates, was observed only during the second half of the study.

**Conclusions:**

Our data demonstrate the utility of SNP typing for monitoring bacterial populations over a defined period in a single endemic setting. We provide evidence for genotype introduction and define a nalidixic acid resistant subclone of *S*. Typhi, which appears to be the dominant cause of severe pediatric typhoid in Kathmandu during the study period.

## Background

*Salmonella enterica *serovar Typhi (*S*. Typhi) is the bacterial agent of typhoid fever. *S*. Typhi is atypical with respect to the majority of *Salmonella *serotypes as it does not have the ability to interact with multiple hosts and is restricted solely to infecting humans [1]. *S*. Typhi has adapted to the human-restricted niche via a number of genetic mechanisms, including the acquisition of virulence factors and a series of gene inactivation events [2-4]. The inability of *S*. Typhi to interact with other hosts appears to have resulted in the organism becoming genetically isolated and dependant on asymptomatic carriage for long-term survival in the human population [5-7].

Typhoid fever remains an important public health issue in many developing countries and predominates in areas with poor sanitation, which aids its transmission and persistence in the human population [1,8,9]. In countries where typhoid is endemic, the adolescent fraction of the population is considered to be at risk [10]. However a recent study in Kathmandu, Nepal demonstrated that natural immunity to *S*. Typhi increased with age, suggesting that the typhoid burden among young children may have been underestimated [11]. We estimate that the prevalence of typhoid fever in some parts of Kathmandu, Nepal is amongst the highest in South Asia [8,12]. A ten year study was recently reported [13], in which 9,124 invasive *Salmonella *isolates were recovered from 12,252 positive blood cultures taken at Patan Hospital in Kathmandu in 1993-2002. The majority of isolates were confirmed as *S*. Typhi. During this retrospective study the overall rate of enteric fever more than doubled between 2001 and 2003, when compared to the three previous years [13]. Multiple drug resistance was not deemed a significant problem in Kathmandu during 1993-2003. However in Kathmandu, as in other parts of Asia, reduced susceptibility to fluoroquinolones increased during this time [14]. Fluoroquinolones are broad-spectrum antimicrobials and include ciprofloxacin, one of the currently recommended and highly effective antimicrobials for the treatment of enteric fever in endemic regions [15]. Resistance to nalidixic acid is a marker for reduced susceptibility to fluoroquinolones, and mutations which induce initial nalidixic acid resistance often precede the evolution of fluoroquinolone resistance [16]. Fluoroquinolones target bacterial topoisomerases, in particular the DNA gyrase protein (GyrA) and inhibit DNA replication. A single nucleotide mutation at either codon 83 or 87 of the gene encoding the GyrA protein (*gyrA*) confers resistance to nalidixic acid in *S*. Typhi [17].

The detection of phylogenetically informative genetic variation is pivotal for the study of pathogen populations circulating in a given area and over time. Many techniques are routinely used to achieve differentiation between bacterial pathogens. However, not all methods provide phylogenetic and genotypic information offering a high degree of specificity, reproducibility and sensitivity. Arguably, only systems that allow a direct comparison of specific nucleotide sequences, such as multi-locus sequence typing (MLST) or single nucleotide polymorphism typing (SNP typing) permit accurate differentiation of a bacterial population [18,19].

Defining the circulating population is particularly challenging for studies of *S*. Typhi, as this organism is monophyletic and sequence diversity is limited [20]. The generation of a rooted phylogenetic tree based upon rare SNPs has permitted a greater understanding and definition of the global population of *S*. Typhi, and provided a potential method for tracking the pathogen in an endemic setting [21]. By assaying SNPs using a high-throughput Sequenom platform, we were previously able to identify several genetically distinct *S*. Typhi haplotypes circulating in an urban area of Jakarta, Indonesia [22]. More recently, we used high-throughput sequencing technologies to discover nearly 2,000 SNPs within the *S*. Typhi population, providing additional loci for SNP typing of clinical isolates [5]. Importantly, the study detected relatively few examples of other forms of genetic variation such as insertions and deletions (<30 in total), which could potentially be used as markers for studying *S*. Typhi populations [5].

In the present study, we have developed a custom SNP array for *S*. Typhi using the GoldenGate platform (Illumina). The array includes 1,500 of the SNPs identified previously in the *S*. Typhi genome [5], providing greater discriminatory power than any previous study of *S*. Typhi and as high as any bacterial SNP typing assay reported to date [23]. As such the array allows the discrimination of distinct bacterial clones co-circulating in a local area, which may be associated with drug resistance or disease severity. Here we have used the array to differentiate subclones of *S*. Typhi causing severe typhoid in children aged 2 months - 12 years, admitted to Patan Hospital in Kathmandu, Nepal, between April 2005 and December 2006 [24].

## Methods

### Study design and sample collection

The study design was as described previously [24]. Briefly, samples were isolated at Patan Hospital, Kathmandu, Nepal. Patan Hospital is one of only two large hospitals in Kathmandu Valley that accepts pediatric referrals and has pediatric inpatients. All children aged 2 months - 12 years admitted to the pediatric ward at Patan Hospital from April 2005 to December 2006 with fever (temperature >38°C) and/or suspected septicaemia were considered for entry into the study. Admission to hospital in this case implies severe disease, considered too ill to be treated as outpatients and requiring treatment with IV antibiotics. For the final six months of the study, a number of patients attending the outpatient clinic were also enrolled for examination. These patients had less severe symptoms, requiring oral administration of antibiotics only.

Demographic and clinical data were collected by local clinical research officers after informed consent was obtained and then were entered into a standardized database. Exclusion criteria were the absence of blood culture specimens collected at admission, hospitalization for any illness within the previous 10 days, and presentation with recurrent wheezing or acute gastroenteritis alone [24].

Invasive *Salmonella *(*S*. Typhi and *S*. Paratyphi A) were the most common bacterial isolates from the study of children with suspected septicaemia. In total, *S*. Typhi was recovered from 62 blood cultures from children with suspected septicaemia, of which 46 were treated as inpatients and 16 as outpatients. Ethics approval for this study was obtained from Oxford Tropical Research Ethics Committee (OXTREC 032-06) and the Nepal Health Research Council.

### Bacterial isolation and identification

Blood culture specimens were obtained from all eligible children by either hospital staff or staff involved in the study. Blood specimens of 1-3 ml were collected from all children before antibiotic administration, whenever possible. The majority of blood culture specimens were collected into commercially prepared Soybean-Casein Digest Broth with Resin blood culture bottles (BACTEC Peds Plus/F; Becton Dickinson), and all were processed manually. Blood culture bottles were incubated at 35°C and were inspected twice daily for turbidity. All samples had sub-culturing performed, irrespective of turbidity, at 12-24 h and on day seven of incubation. Identification of Salmonella isolates were confirmed using API-20E strips (bioMerieux, France) and serotyping was performed using standard Salmonella serotyping methodology (Murex, United Kingdom).

### Antimicrobial resistance testing

The antimicrobial susceptibility patterns to ampicillin, chloramphenicol, co-trimoxazole, ciprofloxacin, nalidixic acid and ceftriaxone were determined by standard disk diffusion methodology. Zone size interpretation was measured according Clinical and Laboratory Standards Institute guidelines [25].

### Nucleic acid isolation

A suspension of an overnight culture of *S*. Typhi was prepared in 1 ml of Hanks Balanced Salt Solution and centrifuged at 16,000 × g rcf in a microfuge for two minutes. Cells were re-suspended in 600 μl of Nuclei Lysis Buffer and DNA was extracted as recommended by the manufacturers guidelines (Wizard genomic DNA extraction kit) (Promega, USA). DNA was re-suspended in 100 μl of rehydration solution and incubated at 65°C for one hour. The DNA was stored at 4°C until required for typing.

### *S*. Typhi SNP typing

DNA samples were quantified using the Quant-IT kit (Qiagen, USA) and concentrations adjusted to 10 ng/μl using nuclease-free water (Ambicon, USA). DNA samples were assayed using custom oligo pools using the GoldenGate (Illumina) protocol. DNA samples were arrayed randomly in a 96-well plate, along with one blank (water) well and control samples (DNA from sequenced Typhi isolates described in [5]). The array design and genotype identification was performed as previously described [26]. The array included targets for over 2,000 loci in the Typhi chromosome and an IncHI1 drug resistance plasmid [5], and two *S*. Paratyphi A-specific SNPs to facilitate identification of mis-serotyped isolates. Sequences surrounding each known SNP were submitted to Illumina for preliminary design in two pools. SNPs within 60 bp cannot be included in the same oligo pool, and SNPs within 10 bp cannot be assayed at all using this method, due to interference with specific primer binding. SNPs with SNP score >0.4 were included in the final assays, comprising two primer pools (200 SNPs were included on both arrays for quality control purposes). Briefly, signals from the GoldenGate assay were analyzed using Illuminus-P as previously described [26,27]. The final analyzed dataset provided high quality allele data for 1,485 chromosomal SNPs and eight plasmid SNPs [26,28]. The full list of SNP loci and alleles used in the analysis is given in additional file 1: SNP table. Owing to the proximity of the SNPs in the *gyrA *gene, these were detected by use of a Luminex xMAP system [29,30].

### Analysis of SNP data

**Figure 1:**
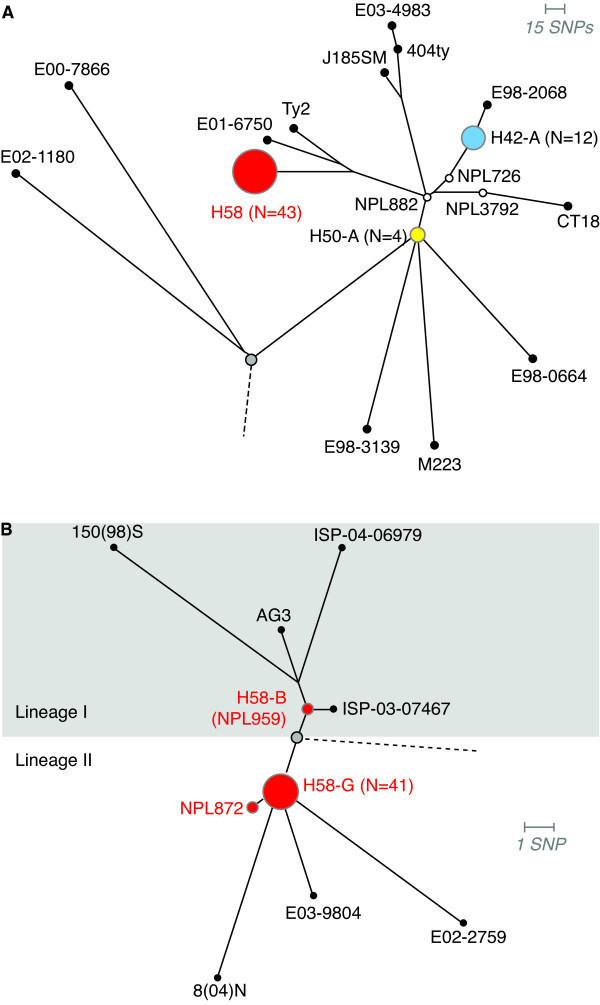
**Phylogenetic trees based upon SNPs, demonstrating the relationship of 62 *S*. Typhi isolates from Nepalese children**. A) Phylogenetic tree showing haplotypes detected in pediatric samples from Nepal (highlighted in red, white, blue and yellow circles). The tree root (representing other *Salmonella *serotypes) is shown in grey. Branch lengths indicate an estimated rate of substitutions per assayed SNP locus, as calculated by RAxML. Length of the scale bar is 0.01 substitutions per site, equivalent to approximately 15 SNPs. B) Zoomed-in view of the H58 group in (A). The root of the H58 clonal group is shown in grey; the dashed line represents the link to the remainder of the *S*. Typhi phylogenetic tree. The division between lineage I and lineage II is indicated using grey vs white background. Leaves of the tree correspond to previously sequenced *S*. Typih isolates; 8(04)N (Vietnam, 2004), E03-9804 (Nepal, 2003), E02-2759 (India, 2002), ISP-03-07467 (Morocco, 2003), ISP-04-06979 (Central Africa, 2004), AG3 (Vietnam, 2004) and 150(98)S (Vietnam, 1998).

SNP alleles at 1,485 chromosomal loci, identified from GoldenGate signals using Illuminus-P [26,27], were concatenated to give a single haplotype string for each strain. The alignment of these haplotype strings was used as input for phylogenetic analysis. Initially, the alignment was analyzed using ModelTest which suggested a general time reversible (GTR) model provided the most appropriate phylogenetic model for this data. RAxML was used to fit 1,000 bootstrapped tree topologies and optimize branch lengths using a GTR model [31,32]. SNP typing provides genetic information only at the specific assayed loci; in this case these loci were mostly determined by whole genome comparison of 19 *S*. Typhi strains as described in [5]. Thus, fitting a tree to this data is analogous to fitting each novel *S*. Typhi isolate into the phylogenetic structure defined previously [5], and the resulting branch lengths reflect genetic divergence only at the assayed SNP loci (Figure 1).

### Statistical analysis

Rates of female sex and in-patient status were compared between haplotype groups using pairwise Pearson Chi-square tests. The distributions of patient and clinical variables for patients infected with *S*. Typhi H58-G and other haplotypes were compared using a two-sample Kolmogorov-Smirnov test. All statistical analysis was performed using the R package for statistical computing http://www.r-project.org.

## Results

### Distribution of *S*. Typhi haplotypes detected

A total of 62 S. Typhi isolates were available for analysis. Of these, 46 came from children admitted to Patan Hospital (severe typhoid requiring inpatient treatment with IV antibiotics) and 16 from children treated as outpatients (less severe typhoid requiring oral administration of antibiotics). The haplotypes detected among these isolates are indicated in Figure 1 and summarised in Table 1. The majority of isolates (70%) were of the H58 haplotype (Figure 1). Haplotype H42, genetically distant from H58 was also common (19% of isolates), while the remaining isolates were from distinct haplotypes (Figure 1a).

**Table 1:** Case parameters of 62 *S*. Typhi isolated from Nepalese children identified by haplotype

	H58 lineage II	H42-A	Other	All
**Isolate details**				
No. isolates	42	12	8	62
Nalidixic acid resistance	42 (100%)	0	0	42 (68%)

**Patient details**				
Mean age	5	4.4	3	4.6
Female sex	20 (49%)	5 (42%)	5 (56%)	30 (48%)
Inpatient treatment	30 (73%)	9 (75%)	7 (78%)	46 (74%)

**Clinical variables (N with data available)**	30	8	5	43
Mean duration hospital stay (days)	8	6	7	7
Fever duration prior to admission (days)	10	9	9	9
Fever temperature at admission (°Celsius)	38.98°	38.68°	38.83°	38.82°
Hepatomegaly	6 (20%)	1 (13%)	1 (20%)	8 (19%)
Splenomegaly	1 (3%)	1 (13%)	0 (0%)	2 (5%)
Acute diarrhoea	8 (27%)	4 (50%)	2 (40%)	14 (32%)

The GoldenGate assay utilized in this study included 44 SNP loci known to be variable amongst the H58 group, these specific SNPs included 39 that were identified during genome sequencing and five loci previously identified in H58-derived haplotypes (see additional file 1: SNP table) [5,21]. These individual SNP loci allow further differentiation of the currently common H58 haplotype and permit a higher degree of sensitivity and resolution. The additional H58 specific SNPs sub-divide the H58 population into two main lineages, I and II, which can be sub-divided further into strain-specific sub-lineages (Figure 1b, SNPs defining these lineages are shown in additional file 2: H58 tree and SNPs). Of the 43 H58 isolates that were identified in this study, 41 were indistinguishable at all 44 intra-H58 loci. The shared haplotype of these strains, referred to hereafter as H58-G, lay in sub-lineage II, and shares recent common ancestry with sequenced strains 8(04)N, E03-9804 and E02-2759 from Vietnam, Nepal and India, respectively [5]. This common ancestry is potential evidence for location-specific development of H58 lineage II *S*. Typhi in typhoid-endemic regions of South Asia.

A single H58 isolate, NPL872, shared a similar haplotype to the 41 identical strains but also harboured an additional SNP that had been previously detected in strain 150(98)S from H58 lineage I (TreB-Asp135, *S*. Typhi CT18 coordinate 4,653,894) (Figure 1b). This could be the result of recombination between different subtypes within the H58 population. However, due to the limited recombination in the *S*. Typhi gene pool, homoplasy seems a more likely explanation, whereby the same mutation has arisen independently in different lineages. A second H58 isolate, NPL959, lay in lineage I and unlike the H58-G group of strains appears to share recent common ancestry with previously sequenced strains AG3, 150(98)S, ISP-03-07467 and ISP-04-06979 from Vietnam, Vietnam, Morocco and Central Africa, respectively (Figure 1b).

The second most common haplotype was shared by 12 isolates, forming a subgroup of the previously defined H42 haplotype, referred to hereafter as H42-A. All strains belonging to the H42-A group were identical at all SNP loci tested. The remaining isolates include four strains belonging to the H50 haplotype (H50-A), and three singletons each belonging to a unique haplotype (Figure 1a).

### *S*. Typhi haplotype is not associated with age, sex or disease severity

**Figure 2:**
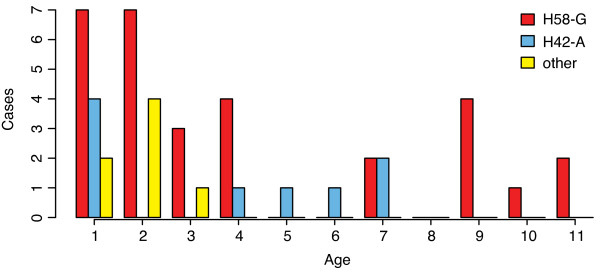
**The distribution of patient ages (years) for infections with each haplotype**. Distributions of the age of patients infected with distinct *S*. Typhi haplotypes.

It is currently unknown if the variation among *S*. Typhi haplotypes or lineages drives observable differences in transmission or clinical presentation of disease syndrome. In this study we found no significant association between *S*. Typhi haplotype and patient age, sex, treatment as an inpatient or disease severity measures for inpatients (Table 1). Haplotype was not associated with inpatient (severe disease requiring hospital admission and IV antibiotics) vs outpatient status. For children admitted as inpatients, *S*. Typhi haplotype was not associated with fever temperature at admission or duration of hospital stay (Table 1). The mean age of children infected with *S*. Typhi of the H58-G haplotype was slightly higher than among those infected with other haplotypes (5.0 years vs 4.4 or 3.0 years for other haplotypes, Table 1), as shown in Figure 2. However the difference in age distributions was not significant (two-sample Kolmogorov-Smirnov test) and is most likely an artefact driven by the high frequency of H58-G strains in this study. During the period of the study, infections in older children were generally less common and thus the chance to observe older children infected with other haplotypes was reduced compared to the more abundant H58-G strains. There does however appear to be a higher level of haplotype diversity among children under four years of age (Figure 2).

### Antimicrobial Resistance

All *S*. Typhi isolates were tested for resistance to ampicillin, chloramphenicol, co-trimoxazole, gentamicin, ciprofloxacin, ceftriaxone and nalidixic acid at the time of initial isolation. All isolates were susceptible to the former six drugs, while only the 42 isolates of H58 lineage II (including NPL872) were resistant to nalidixic acid (Table 2).

**Table 2:** Nalidixic acid resistance in 43 H58 *S*. Typhi isolates

H58 lineage	H58 subgroup	Nalidixic acid	GyrA SNP	Treatment	N1
II	G	resistant	Phe83	inpatient	30
II	G	resistant	Phe83	outpatient	11
II	G*	resistant	Phe83	outpatient	1
I	B	sensitive	wildtype	inpatient	1

Multidrug resistant (MDR) *S*. Typhi (resistant to ampicillin, chloramphenicol and co-trimoxazole) has been reported previously in Nepal and is usually associated with the presence of a large IncHI1 plasmid [9,33,34]. All oligos in the GoldenGate assay targeting IncHI1 plasmid SNPs failed to generate fluorescent signals in Nepalese isolates, while generating signals for control isolates that contain IncHI1 MDR plasmids (CT18, E03-9804, ISP-03-07467 and ISP-04-06979 [5]). This is consistent with the lack of resistance phenotypes among our Nepalese isolates and indicates that the IncHI1 resistance plasmids common to *S*. Typhi were absent from the those responsible for typhoid in Kathmandu children during the period of our study.

Nalidixic acid resistance was restricted only to the H58-G group and closely related isolate NPL872, with all 42 isolates demonstrating resistance using the disc diffusion method (Table 1). All H58 isolates were tested for six known *gyrA *mutations: Pro83, Phe83, Tyr83, Asn87, Tyr87 and Gly87 [21]. All H58-G isolates and NPL872, each of which displayed nalidixic acid resistance, harboured the Phe83 mutation but were wildtype at codon 87 (Table 2). The lone H58 lineage I isolate NPL959 was nalidixic acid sensitive and carried wild type alleles at codons 83 and 87 in the *gyrA *gene (Table 2).

### Seasonal Differences in *S*. Typhi Infections

**Figure 3:**
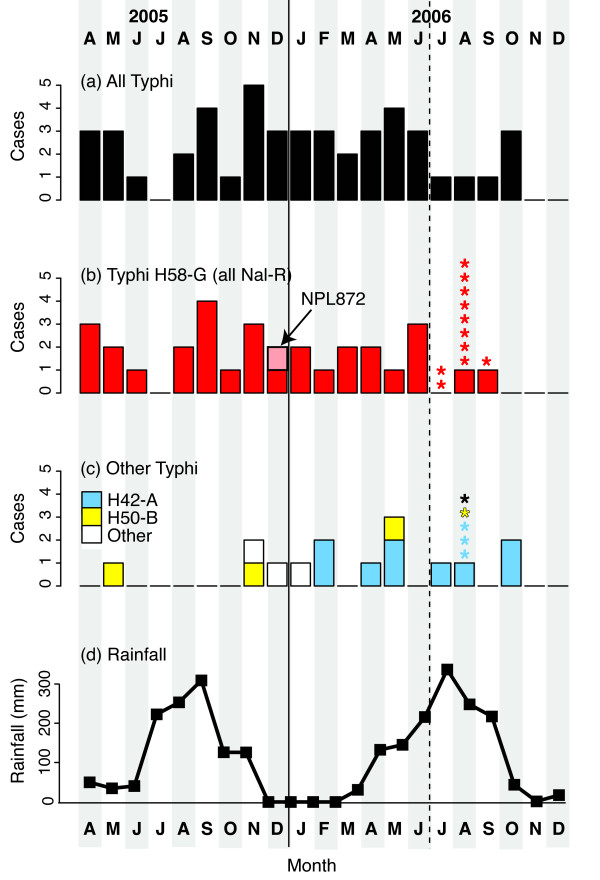
**Temporal distribution of *S*. Typhi from 62 genotyped organisms from Kathmandu**. A) all inpatient cases, B) all infections with H58-G genotype *S*. Typhi organisms, C) patients infected with other *S*. Typhi haplotypes, including H42-A and H50-A, D) seasonality of rainfall, taken from the nearest weather station (Kathmandu Tribhuvan International airport). Bars = inpatient cases; Stars = outpatient cases; inpatient cases were only included in last six months of study period, distinguished by the dashed line.

The distribution of typhoid cases during the course of the study is shown in Figure 3. It is believed that the rate of *S*. Typhi infection in temperate regions peaks during the warmest and wettest time of the year [35]. However the incidence of typhoid infections requiring hospitalization was evenly distributed throughout the study period (mean 2.5 cases per month). During the final six months of the study, *S*. Typhi isolates from pediatric outpatients were also collected. The majority of these cases occurred in August, the middle of the wet season (see Figure 3). Thus, there may be a general increase in the incidence of typhoid fever among children during the wet season, but this was not associated with an increase in severe typhoid cases requiring hospitalization, which remained constant throughout the year.

Notably, the temporal pattern of infection differed among *S*. Typhi haplotypes (Figure 3). Hospitalization of patients infected with H58-G *S*. Typhi occurred at a constant rate during the course of the study (mean 1.4 cases per month, Figure 3). However H42-A *S*. Typhi was not identified until the second half of the study (Figure 3c, blue), after which point this haplotype was detected among inpatients at a mean rate of 0.8 cases per month. Hospitalizations due to infection with other *S*. Typhi haplotypes occurred throughout the study, at a mean rate of 0.33 cases per month. The distribution of haplotypes among isolates collected from outpatients during the last six months of the study is shown in Figure 3b and 3c. The distribution of haplotypes was similar for inpatients and outpatients, as indicated in Table 1.

## Discussion

*S*. Typhi is a monomorphic bacterium which is spread solely in human populations: it is recently evolved and genetically isolated and thus sequence diversity within this bacterial population is highly limited [5,20,21]. To distinguish and identify commonality between strains, an assay is required that can identify a limited number of nucleotide changes on a genome wide scale. Here, we describe the first high-throughput SNP-based typing study of *S*. Typhi strains circulating in Kathmandu, Nepal. The development of the GoldenGate assay, assessing 1,500 single nucleotide base changes around the genome, permits a degree of sensitivity and specificity that has never previously been achieved in a monomorphic bacterial population, and also allows true phylogenetic comparisons. Our findings from this initial investigation using a whole genome interrogation of *S*. Typhi shows a limited number of clones circulating in a local population, with one predominating.

Typhoid fever is an ongoing public health issue in some countries in Southern Asia and Nepal (especially Kathmandu) is known to have a high burden [8,12,13]. Studying the structure of the local population of *S*. Typhi is particularly important given the recent increase in strains which are resistant to nalidixic acid and have reduced susceptibility to fluoroquinolones [36,37]. Such strains prolong fluoroquinolone treatment regimes; even more alarming are recent reports of fully fluoroquinolone resistant *S*. Typhi [38,39]. Recent genomic studies have provided a greater understanding of the organisms that cause enteric fever (*S*. Typhi and *S*. Paratyphi A) [3,40] and comparative studies within the *S*. Typhi population have identified variant loci that facilitate more informative molecular typing of the organism [5,21]. The SNP typing approach presented here is reproducible, robust and able to provide phylogenetic information with which to identify clonal expansion or replacement.

The present study design does however pose some limitations, which affect interpretation of the resulting data. The study was not population-based, rather it represents a tiny snapshot of all the typhoid cases in the community, focusing on children with severe typhoid aged twelve years and under. There was asymmetrical sample collecting over the period of surveillance, whereby outpatient samples were included only for the final six months of the study. In particular, it should be noted that the majority of isolates studied (46) were from severe typhoid cases with a history of fever for a mean duration of nine days prior to admission to hospital, requiring hospitalization for a mean length of seven days and treatment with IV antibiotics. The majority of typhoid cases are treated at outpatient centres, local health care centres or at home after purchase of antimicrobials, which are widely available and subject to uncontrolled use. Thus the samples analysed here may not be representative of the whole of *S*. Typhi infections occurring in the human population in Kathmandu.

In a previous descriptive study of 408 typhoid patients in the same setting, the median age of infection was 17 years, suggesting that childhood typhoid in Kathmandu is a relatively small fraction of the total disease burden [8,12]. In the present study we found no association between age and haplotype; however further investigation is required to study the haplotype distribution in the broader population of typhoid fever patients in Kathmandu. There was however greater haplotype diversity among children under four years of age (Figure 2). This coincides with lower levels of natural immunity previously observed in this age group, which might result in susceptibility to a broader range of haplotypes compared to older children who have increased immunity and may be susceptible to a narrower range of more virulent haplotypes [11]. However a larger study of typhoid patients will be required to confirm this hypothesis.

Understanding the distribution of strains over a longer period may help to define transmission patterns and the distribution of circulating haplotypes of *S*. Typhi in this location. A prospective study of severe typhoid infections may also aid understanding of disease severity related to bacterial genotype. The observation that specific genotypes cause severe disease has been made using Pulsed Field Gel Electrophoresis (PFGE) but not confirmed by means of newer sequence based typing [41].

Despite the limitations of this work there are some valuable observations from the findings. Firstly, the *S*. Typhi H58 haplotype was the principal strain detected within the study population; this is also the case in Vietnam and other endemic locations [21]. This finding is consistent with the hypothesis that the H58 strain is a dominant clone that spreads easily within and between human populations and has become efficiently maintained within those populations. Furthermore, the highly dominant H58 clone has been associated with nalidixic acid resistance, due to resistance-conferring mutations in the *gyrA *gene [21]. Our data validates this observation, as all H58 lineage II isolates carried *gyrA*-Phe83 mutations and these were the only isolates demonstrating nalidixic acid resistance. This could be indicative of positive selection and dissemination of a single nalidixic acid resistant H58 clone at high frequency in Kathmandu. The continuing use of fluoroquinolones, possibly at sub-optimal doses within the community, may continue to aid the ongoing selection of these organisms.

Patients with typhoid caused by organisms belonging to the H58 group were seen at a constant rate throughout the duration of the study. Strain types additional to H58 were also evident, but less common and included H50-A and H42-A. Of particular interest is the detection of the H42-A haplotype, the second most common type, only in the second half of the study period. There are several possible explanations for this. There may be fluctuation in environmental factors that differentially affect transmission of certain strain types, thus some genotypes may only appear (and then presumably disappear) transiently. This may be as simple as stochastic variation in the relative frequencies of Typhi strains within local water sources, or strain-specific variation in survival under different environmental conditions including fluctuations in temperature or osmolarity. It is also possible that the H42-A genotype was introduced into the study population from an exogenous source during the course of the study. The infection source in these patients is unknown, so we were not able to determine any additional links between these cases besides their timing. Long-term carriage of invasive *Salmonella *is known to be relatively common in this setting [6], and it could be that all the H42-A cases were infected from the same source or had been in contact with a carrier shedding this organism. Long-term epidemiological studies incorporating genotyping will be needed in order to investigate the issue of strain introduction and/or transient infection cycles.

## Conclusions

Our findings demonstrate the circulation of multiple *S*. Typhi strains in Nepalese children during a two-year period, and in particular the dominance of a single clone associated with reduced susceptibility to fluoroquinolones. The novel SNP-based methodology used represents an important tool in molecular epidemiological studies and as our understanding of the biology and diffusion of the organism increases it will permit the long-term monitoring of strains circulating in specific locations with high transmission of *S*. Typhi.

## Competing interests

The authors declare that they have no competing interests.

## Authors' contributions

Study design and interpretation: GD, AJP, DFK, DRM, JF, SB, JP, KEH. Sample collection, bacterial isolation and DNA extraction: SD, BB, NA, ST, ASP. Resistance testing: DM. GyrA SNP typing: YS. Data analysis: KEH. Manuscript drafting: KEH, SB, GD. All authors read and approved the final manuscript.

## Pre-publication history

The pre-publication history for this paper can be accessed here:

http://www.biomedcentral.com/1471-2334/10/144/prepub

## Supplementary Material

Additional file 1 **SNP table**. Details of all chromosomal SNPs used in this study, including position in the *S*. Typhi CT18 genome [EMBL: AL513382], alternative alleles, assignments to two oligo pools for the GoldenGate assay, source of SNP discovery and whether the SNP is variable among H58 strains. Click here for file

Additional file 2 **H58 tree and SNPs**. Phylogenetic tree of H58, showing which SNPs define each branch. SNPs are labelled with their position in the CT18 genome [EMBL: AL513382], alleles and other details are given in Additional file 1 - SNP table. Click here for file
