# Supplementary figures and images for: High-throughput bacterial SNP typing identifies distinct clusters of Salmonella Typhi causing typhoid in Nepalese children
Source: BMC Infect Dis. 2010 May 31;10:144. doi: 10.1186/1471-2334-10-144 (PMC2897797; doi:10.1186/1471-2334-10-144)

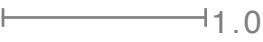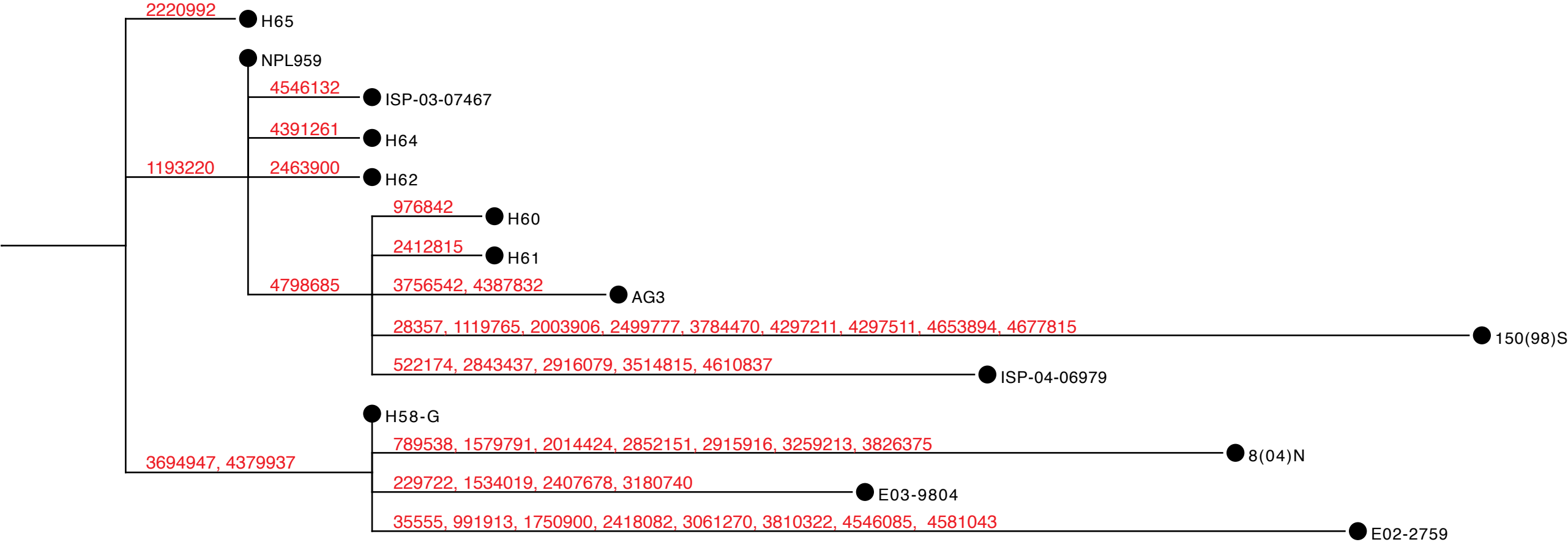

Supplement: Additional file 2 — H58 tree and SNPs. Phylogenetic tree of H58, showing which SNPs define each branch. SNPs are labelled with their position in the CT18 genome [EMBL: AL513382], alleles and other details are given in Additional file 1 - SNP table. [file 1471-2334-10-144-S2.PDF]
